# The Potential Antimicrobial Action of Human Mucin 7 15-Mer Peptide and Its Metal Complexes

**DOI:** 10.3390/ijms23010418

**Published:** 2021-12-30

**Authors:** Anna Janicka-Kłos, Hanna Czapor-Irzabek, Tomasz Janek

**Affiliations:** 1 Department of Inorganic Chemistry, Wroclaw Medical University, Borowska 211A, 50-556 Wroclaw, Poland; 2 Laboratory of Elemental Analysts and Structural Research, Wroclaw Medical University, Borowska 211A, 50-556 Wroclaw, Poland; hanna.czapor-irzabek@umw.edu.pl; 3 Department of Biotechnology and Food Microbiology, Wroclaw University of Environmental and Life Sciences, 51-630 Wroclaw, Poland; tomasz.janek@upwr.edu.pl

**Keywords:** MUC7 (MG2), antimicrobial peptides, salivary peptides, Cu(II)/Zn(II) coordination properties

## Abstract

Mucin 7 (encoded byMUC7) is a human salivary protein that has a role in the natural immune system. Fragments of mucin 7 exhibit antimicrobial activity against bacteria and yeast. Although the antimicrobial properties of peptides have been known and studied for decades, the exact mechanism of action of antimicrobial peptides (AMPs) is still unclear. It is known that some AMPs require divalent metal ions to activate their activity. Herein, we investigated three 15-mer MUC7 peptides, one of which (mother peptide, sequence, L3) is a synthetic analog of a fragment naturally excised from MUC7 (with His3, His8, and His 14) and its two structural analogs, containing only two histidine residues, His3, His13 and His8, His13 (L2 and L1, respectively). Since there is a correlation between lipophilicity, the presence of metal ions (such as Cu(II) and Zn(II)) and antimicrobial activity of AMP, antimicrobial properties of the studied peptides, as well as their complexes with Cu(II) and Zn(II) ions, were tested for activity against Gram-positive (*Enterococcus faecalis*, *Staphylococcus epidermidis*) and Gram-negative (*Escherichia coli*, *Pseudomonas aeruginosa*) bacteria and fungi (*Candida albicans*). The results were correlated with their lipophilicity. Coordination and thermodynamic studies (potentiometry, UV-Vis, CD) revealed the formation of mainly mononuclear complexes in solution for all studied systems with different stability in the physiological pH range.

## 1. Introduction

Nowadays, when the body is exposed to newer, more malicious microbes, and multi-drug resistance is a fact, it is essential to search for new, effective drugs.

In recent years, the trend of researching the antimicrobial properties of naturally occurring cationic particles (CAMPs, AMPs), such as proteins or their enzymatically cut fragments, has developed quite strongly. Additionally, a large group of studies focuses on interactions of these molecules with metal ions which can occur in living organisms.

Such molecules include antimicrobial peptides (AMPs). The mechanism of cell membrane damage generally involves two steps. First, the positively charged AMPs selectively bind onto the surface of the negatively charged bacterial cell membranes [1]. AMPs can kill bacteria by direct interaction with their membrane [2], as in the barrel-stave, toroidal and carpet model [3], or without changing the membrane integrity through inhibiting important pathways inside the microbe cell such as DNA replication, RNA transcription, or protein synthesis [4,5,6,7].

Among AMPs, there are some peptides derived from salivary proteins. Among them are mucins glycoproteins with a unique structure and properties, present on all mucosal surfaces in the body [8,9,10]. It is also well documented that these proteins play a complex and important protective role for living organisms [10,11].

While the first place where the body protects against microbes is the oral cavity, the process of destruction of pathogens could occur as a result of interaction with components of saliva, including the protein mucin 7 (encoded by MUC7) and its proteolytic cleavage fragments [12,13,14].

Since Zn(II) and Cu(II) ions are crucial for the survival and virulence of bacteria and fungi, and because of the rapid emergence of multi-drug resistant pathogens and/or the relative lack of new effective antibiotics, the development of new metal ion-based therapies that can effectively inhibit the multiplication of pathogens is strongly considered. While the imbalance of metals caused by metal ion chelation may inhibit the proliferation of pathogens [15], the direct cytotoxic effect of the formed chelate compound seems to be of great interest for research.

It has been reported that the activity of CAMPs might be influenced by interactions with metal ions, e.g., Zn(II) and Cu(II) [16,17], that directly or indirectly affect their mechanism of action [16,18,19]. The study of the antibacterial properties of metal complexes with antimicrobial peptides has become the subject of intensive research into the search for effective drugs [20]. Interestingly, a lot of isolated, natural AMPs possess an ACTUN motif which is a very effective Cu(II) binding site. However, the role of copper ions in the mechanism of action of these peptides seems to be still undetermined. Peptide systems reveal various mechanisms of metal ion binding which strongly depend on the amino acid sequence of a ligand, e.g., presence and location of a histidine residue. Taking all these facts into account, we hypothesize that the metal complexes of the 15-mer peptide derived from MUC7, which contains the ACTUN motif and another two His residues, can form stable complexes with metal ions and exert a significant positive effect on the increase in antimicrobial activity compared to the peptide itself.

In our previous article, we reported that the 12-mer derived from the human MUC7 protein and their metal complexes is active against Gram-positive bacteria (*Enterococcus faecalis* and *Staphylococcus epidermidis*) [21]. Since fragments of different chain lengths are formed during enzymatic protein cleavage, we decided to study longer 15 amino acid fragments derived from the fragmentation of mucin 7 protein. A further aim of the study was to reveal the effect of peptide elongation and the presence of another His residue in the amino acid sequence, which may affect the process of metal ion coordination, so the alanyl analogs of the original 15-mer were also investigated.

## 2. Results and Discussion

### 2.1. Study of Antimicrobial Activity In Vitro

The antimicrobial activities of three peptides KSAFELPHYGLLAHQ (L1), KSHFELPAYGLLAHQ (L2), and KSHFELPHYGLLAHQ (L3) and their metal(II) complexes against *E. faecalis*, *S. epidermidis*, *E. coli*, *P. aeruginosa*, and *C. albicans* were evaluated. As presented in Table 1, all tested peptides and their metal(II) complexes were more effective against Gram-positive bacteria. The strain most susceptible to the compounds tested was *S. epidermidis*. Minimal inhibitory concentrations (MICs) of *S. epidermidis* strain were 132 µM for peptide L1, 147 µM for peptide L2, and 210 µM for L3 in the analysis performed in LB medium. Regarding the human pathogens, bacteria *E. faecalis* (Gram-positive) and *E. coli* (Gram-negative), and yeast *C. albicans*, L1 and L2 peptides were able to completely inhibit the growth (MIC values ranged between 145 and 469 μM). The metal(II) complexes also showed activity against bacteria and fungi (Table 1), in a lower concentration. As compared with the L1 peptide, L1/Cu(II) (MIC = 105 μM), and L1/Zn(II) (MIC = 85 μM) showed improved potency against *S. epidermidis* (Table 1), indicating that the complexing of the copper and zinc increased the peptide activity. The MIC breakpoints were obtained, lower than those for MUC7 12-mer peptides [21] that treat *E. faecalis* (Zn(II) and Cu(II) complexes, as well as MUC7 12-mer alone), *S. epidermidis* (MUC7 12-mer peptides and their metal complexes), and *E. coli* and *P. aeruginosa* (MUC7 12-mer peptides and its complexes). Finally, the results obtained for *C. albicans* clearly shows that 15-mer MUC7 peptides and complexes with Cu(II) and Zn(II) present competitive values for 12-mer MUC7 peptides [21]. Several AMPs require metal ions for their antibacterial and antifungal activity. Often, metal coordination triggers various metal−ligand or charge−charge interactions that may lead to the destabilization or permeabilization of the cell wall [16,17,22]. In our opinion, 15-mer MUC7 peptides and their metal(II) complexes with moderate hydrophobicity can damage the cell wall or cell membrane structure of microorganisms, which results in cell lysis or the formation of transient pores and the transport of peptides and metal-peptide complexes inside the cell; this property enables them to interact with intracellular targets.

### 2.2. Metal Complexes

**Figure 1:**
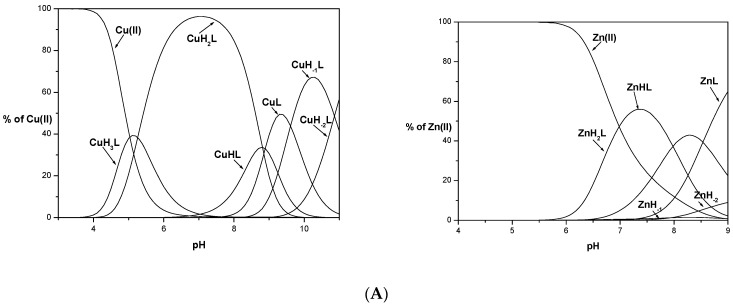

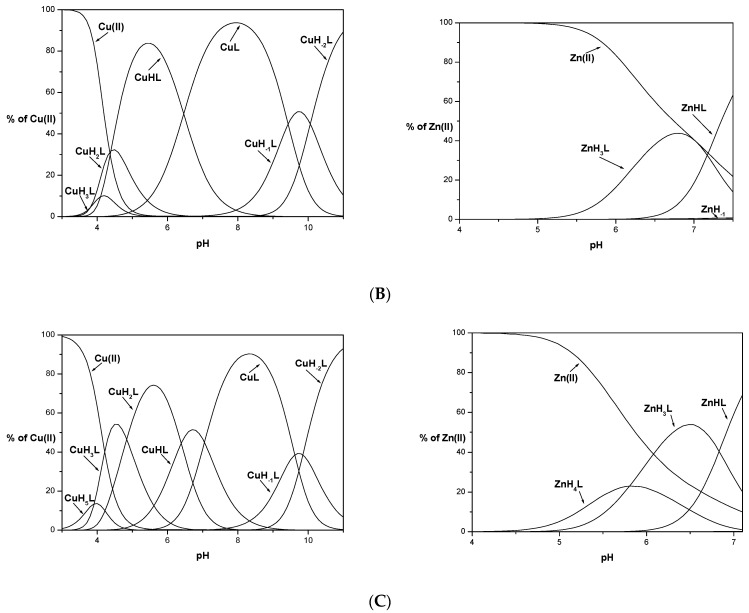
Species distribution of metal (Cu(II) and Zn(II)) complexes of KSAFELPHYGLLAHQ (**L1**), (**A**) KSHFELPAYGLLAHQ (**L2**), (**B**) and KSHFELPHYGLLAHQ (**L3**), (**C**). M(II) to peptide molar ratio 1:1.1; T = 25 °C; I = 0.1 M; [M(II)] = [L1–3] = 0.001 M. Zn_-1_H and Zn_-2_H represent hydrolysis of Zn(II) ions.

Potentiometric studies revealed that all studied ligands form equimolar complexes in the studied pH range (3–11). Distribution profiles for Cu(II) and Zn(II) complexes calculated based on titrations are depicted in Figure 1. Complexes of L1 with Zn(II) were precipitated in the experimental concentration used for potentiometry above pH 9, while for L2 and L3 complexes were insoluble above pH 7. For copper complexes, precipitation was not observed at any experimental pH value.

To confirm the stoichiometry of the formed complexes, mass spectrometry was performed at various pH values (spectra are shown in Supplementary Materials, Figures S1–S9). Almost all observed signals in the MS Zn(II)/L(1–3) spectra were assigned to the appropriate ligand ions or metal complexes. Only mononuclear species with a low relative intensity to the signals assigned to the ligands were observed.

In all the mass spectra of Cu(II)/L1–3 and Zn(II)/L1–3, we can observe the prevailing signals which come from the equimolar Cu(II) complex and Zn(II) complexes, respectively. Signals visible in both spectra come from sodium and potassium adducts of ligands or of their metal complexes. Simulated isotopic patterns of formed metal complexes are in perfect agreement with the experimental ones that confirm the formation of mononuclear complexes with all studied ligands L1, L2, and L3.

Protonation and stability constants for studied peptides and their Cu(II) and Zn(II) complexes are collected in Table 2. Spectroscopic parameters for main Cu(II) complexes are shown in Table 3. Electrospray ionization mass spectrometry (ESI-MS) results are presented in Tables S1–S4 (Supplementary Materials).

All studied ligands have free N-terminal nitrogen and a minimum of two imidazole nitrogens which are potentially anchoring sites for metal ions in the coordination process. In the experiment, during potentiometric titration, as a result of increasing the pH of the solution, various complex forms were formed, in which the metal ion was coordinated with a different number of nitrogen donor atoms. The final donor set involved in metal ion coordination comprises the N-terminal nitrogen atom, imidazole nitrogen(s) plus a set of deprotonated amine donors for Cu(II). Since the studied peptides include two (L1 and L2) and three (L3) histidyl residues in their sequences, which are His8 and His14 for L1, His3 and His8 for L2, and His3, His8, and His14 for L3, different models of metal ion coordination are possible. To elucidate the influence of the presence of His 8 in the sequence of the MUC7 (L3) fragment on its coordination ability, His3 and His8 were replaced during synthesis with an alanine residue in L1 and L2, respectively.

15-mer ligands L2 and L3 have the same amino acid sequence at the N-terminal of the peptide so they indicated an identical coordination model in the studied conditions with slightly different stability of formed complexes following the results obtained for the shorter 12-mers [21].

Based on potentiometric results, L1 and L2 behave like H_7_L while L3 behave like H_8_L acid. The lower pKa (dissociation constant) refers to the deprotonation of the carboxylic group of the C-terminal and glutamic acid (Table 2). pKa values 39.73, 33.75 (L1), 39.84, 34.22, 27.50 (L2), and 45.21, 39.81, 33.47 (L3) are related to protonation steps of His residues: His8, His14 (L1), His3, His14 (L2), and His3, His8, His14 (L3), respectively. pK values of deprotonation of the N-terminal nitrogen atom are 7.59, 7.42, and 7.36 for ligands L1, L2, and L3, respectively, and precede the deprotonation of the Tyr (9.44, 9.58, 9.35) and Lys (9.92, 10.50, 10.01) residues present in the amino acid sequences of the studied peptides.

Comparing the coordination process, L1 is a ligand without an albumin-like motif, which significantly influences the way of its metal ion coordination relative to the other ligands, L2 and L3. The first measured Cu(II) complexes are CuH_3_L for H_7_L (L1, L2) ligands and CuH_5_L for H_8_L (L3) (Figure 1). These forms correspond to 2N type complexes with a metal ion bound via the N-terminus of the peptide and N_im_ of His residue for L1 and L2 {N_NH2_, N_im_} and 1N {N_NH2_} or {N_im_} for L3. Subsequent deprotonations of the ligands in the complexes are caused by binding another N_im_, probably from His14 (CuH_3_L → CuH_2_L) in Cu(II)/L1, the first amide nitrogen (N^-^) (CuH_3_L → CuH_2_L) in Cu(II)/L2, and two nitrogens, the imidazole nitrogen atom of His3 and amide nitrogen (CuH_5_L → CuH_3_L) in Cu(II)/L3 in a cooperative manner. Another formed complex species is a result of coordination with an amide nitrogen atom in all studied systems, CuHL, for L1 (with logβ = 19.09), L2 (logβ = 25.98), and CuH_2_L, for L3 peptide (31.25), respectively. For Cu(II)/L1, two types of complexes are possible: {N_NH2_, N_im_, 2N^−^} or {2N_im_, 2N^−^}, while the second type of coordination is more likely on account of sterility, which is confirmed by computational study and a high pK value of 8.81. As the pH of the solution increases, the second His undergoes deprotonation, His14 for L2 with CuL formation, and His8 or His14 for L3, in a CuHL complex. The third His residue present in L3 undergoes another deprotonation and finally, CuL for L3 is formed. All these changes are well confirmed by obtained spectroscopy parameters, with the maximum wavelength for bands 520 nm and 519 nm in UV-Vis, which corresponds to 4N coordination in Cu(II)/albumin-like peptide complexes [23,24]. The last two complex species, CuH_−1_L and CuH_−2_L, for all studied systems are associated only with deprotonation of the Tyr and Lys residues. This is confirmed by the lack of changes in the values of spectroscopic parameters. The slight differences are probably related to the steric effect that occurred due to the deprotonation process.

In comparison to the shorter 12-mer peptide ligand (lacking His14) [21], bands around 406–417 nm are missing in CD spectra for L1. It may suggest a slightly different pattern of complex formation for this ligand; possibly the first {N_NH2_, N_im_} donor set is involved in metal ion coordination. After further deprotonation 3N {N_NH2_, 2N_im_} and 4N {N_NH2_, 2N_im_, N^−^} complexes are formed. Above pH 8, when the second amide nitrogen is deprotonated, the N-terminal nitrogen leaves the first coordination sphere in favor of amide nitrogen, {2N_im_, 2N^−^}. The maximum wavelength for the band observed in UV-Vis spectra at 520 nm confirms that the 4N complex is present [25,26]. Moreover, an additional band appears in the CD spectra at 532 nm, suggesting the second amide nitrogen atom in the coordination sphere [26], which is in a good agreement with data for {2N_im_, 2N^−^} obtained for peptides with two His residues (H-X-X-X-H) [27]. This type of complex could also be confirmed by the absence of the band around 340 nm, which corresponds to the coordination of the His residue in the apical position [21,28]. Moreover, the comparison of the CD spectra shows that there is no band at the length of about 406–417 nm in the tested Cu(II)/L1 system, which suggests a different way of coordinating the copper ion by the 15-mer and the 12-mer [21]. This might be the effect of the formation of two six-membered chelate rings that are sterically more stable than rings in the N-terminal part of the peptide. This hypothesis could also be supported by the logβ values of formed complexes of Cu(II)/L1, which are higher than for Cu(II)/12-mer lacking His14 [21].

**Figure 2:**
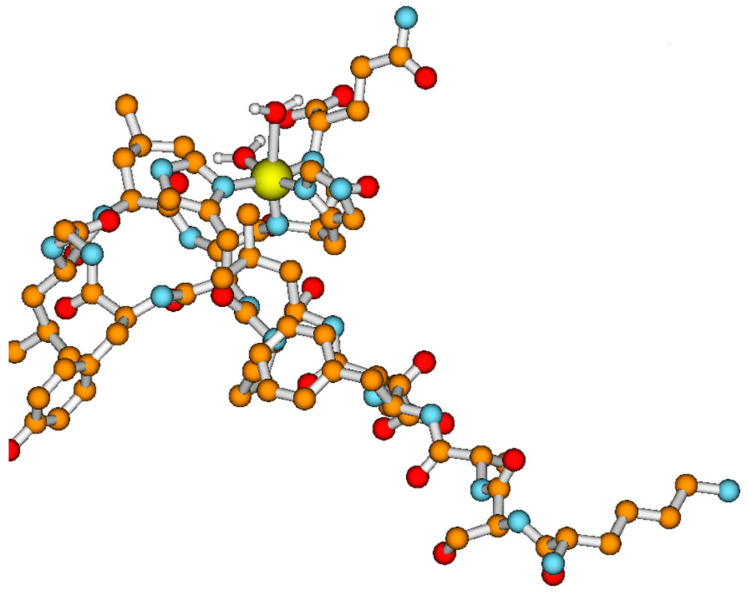
Lowest-energy conformer of CuL form of L1 (KSAFELPHYGLLAHQ) peptide obtained using theoretical calculations. All peptide hydrogen atoms are removed for the sake of clarity. Copper is shown in yellow, nitrogens in cyan, oxygens in red, carbon atoms in orange, and hydrogens in white.

We were able to obtain only the {2N_im_, 2N^−^} CuL stable complex, and the lowest energy conformer for its species is described below. The complex structure is characterized by structural parameters which are in agreement with data obtained from X-ray diffraction measurements collected in the CSD database [29]. As presented in Figure 2, the coordination sphere of the copper ion is composed of two deprotonated amide nitrogen atoms and two imidazole rings of the histidine residue. Two molecules of water are also present. The Cu-N amide distances are 1.8 Å and the distances between imidazole nitrogen and Cu(II) ion are almost equal to 2.0 Å. Metal ion and nitrogen atoms are located in the same plane.

For copper ions binding by ligands L2 and L3, the scheme of the coordination mode is almost identical and is compatible with the coordination of Cu(II) ions by albumin-like peptides. The metal ion is anchored on the N-terminal and N imidazole atom of the His3 residue. Subsequent deprotonations of ligands in the Cu(II)/L2 and Cu(II)/L3 complexes as a result of increasing the pH of the solution lead to the cooperative binding of two amide nitrogen atoms. The result is a stable complex with the 4N type coordination mode and the {N_NH2_, N_im_, 2N^−^} donor set.

Interestingly, the stability of comparable Cu(II) complexes, with the same coordination sphere, formed by L2 and L3, are slightly different. The difference is the number of deprotonated His residues present in the ligand sequences. Copper species formed by L2 are more stable than their L3 ligand counterparts. This is most likely related to the steric effects occurring during the deprotonation of the second His residue in the L3 ligand. It might be that chelating ligand L2, which does not have His8, wraps the metal ion more tightly by the C-terminal part of the peptide than L3, increasing its stability.

**Figure 3:**
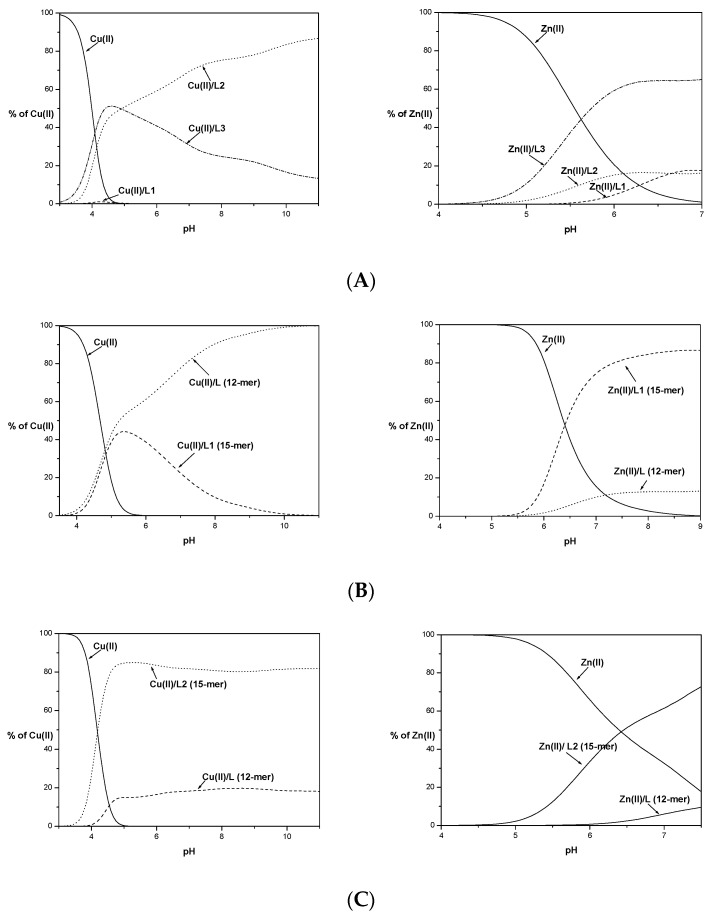

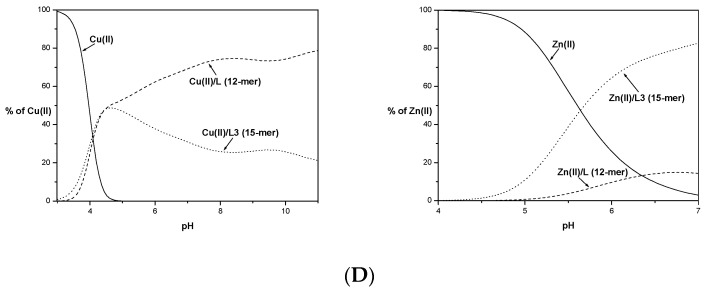
Competition diagram between Cu(II) and Zn(II) complexes of 15-mers: L1, L2, and L3 (**A**) and 15-mers with 12-mers (**B**–**D**), describing complex formation at different pH values in a hypothetical situation. Calculations are based on binding constants from Table 2 and [13]. M(II) to peptide molar ratio 1:1.1; T = 25 °C; I = 0.1 M; [M(II)] = [L1–3] = 0.001 M.

The graph of metal ion binding competition by the tested ligands shown in Figure 3 indicates that Cu(II) binding by the albumin-like motif is unequaled in terms of binding by the two histidyl residues located in the C-terminal part of the ligand L1.

Moreover, higher numbers of histidyl residues do not improve the stability of the complex compounds formed. Ligand L3 is slightly more effective in binding Cu(II) ions only in the initial range of the tested pH (Figure 3A). Above pH 5, its effectiveness declines in favor of the L2 ligand lacking one histidyl residue in its primary structure. The observed destabilization due to the presence of the His residue at position 8 is most likely due to the steric effect of the imidazole ring, preventing the protection of the coordination sphere by the ligand peptide chain.

Competition graphs of the metal coordination ability for studied 15-mer peptides and the His14-deficient 12-mer peptides [21] are shown in Figure 3B–D.

The elongation and insertion of an additional histidyl residue to the sequence compared to the 12-mers increased the stability of the complex species formed only in the case of the L2 ligand. The L1 and L3 peptides up to pH 5 form complexes with similar stability to their His14-deficient 12-mers. Above this pH, the Cu(II)/12-mer complex forms tend to be more stable than L1 and L3 studied herein, most likely due to the formation of smaller chelate rings with greater stability.

L2 starts binding to copper(II) ions in a lower pH value than its 12-mer counterpart (Figure 3C). In both cases, the metal ion binding occurs at the N-terminus of the peptide. The elongation and presence of an additional His residue, stabilizing the resulting Cu(II)/L2 forms, has an impact on the stability of the formed complexes. Stabilization could also be caused by interactions with the side chains of Leu or Gln residues. The exact effect of the presence of these amino acid residues in peptide sequences on the stabilization of complexes with metal ions should be further investigated.

The coordination mode to the Zn(II) ions by the peptide ligands usually involves N-terminal nitrogen, imidazole nitrogens, and carboxylic oxygens. While studied ligands possess a different number of His residues and their localization in the amino acid sequence, the coordination sphere of formed complexes might be slightly different in each system, Zn(II)/L1, Zn(II)/L2, and Zn(II)/L3. The number of His zinc complexes with L1 and L2 should be identical and the relative position of histidyl residues to the N-terminal group should play the main role in the stability of formed complex species.

The coordination of zinc ions with the L1 ligand begins at pH around 5.5 with the formation of the ZnH_2_L complex. The maximum content of this species in the solution is 50% at a pH of around 7.5 (Figure 1). The obtained values of deprotonation constants suggest that this complex is formed by the coordination of the metal ion through the nitrogen atom of the N-terminal amino group and two imidazole nitrogen atoms of the histidyl residues present in the 8th and 14th positions. The C-terminal carboxyl group and glutamic acid have already been deprotonated. The appearance of another complex form, ZnHL, occurs as a result of the deprotonation of the water molecule (pK = 7.98) located in the metal ion coordination sphere. The last calculated complex species, ZnL, is formed at around pH 7.0 due to the deprotonation of another water molecule (pK = 8.72). This coordination model is preferred because the amide nitrogen atoms of the peptide bond do not participate in the binding of zinc ions [30]. It is consistent with the results obtained in studies on peptide ligand systems with zinc ions [31]. During the experiment precipitation of the complex was observed in the studied system above pH 9, which made it impossible to determine the stability constants of successive species formed after deprotonation of the tyrosine and lysine side chains. However, the existence of complexes with metal ions at higher pH values was confirmed by the results of MS studies (Table S2, Supplementary Materials).

For L2 and L3 ligands, precipitations of the formed Zn(II) complexes during potentiometric titration were observed at pH 7.5 (L2) and pH 7 (L3). Based on the experimental curves, it was possible to determine stability constants only for some complex species (ZnH_3_L and ZnHL for L2 and ZnH_4_L, ZnH_3_L and ZnHL for L3 ligand). The obtained values of the stability constants do not allow for exact determination of whether all the nitrogen atoms of the imidazole His residues are involved in the coordination of the metal ion. Based on the competition diagram, Figure 3A, it could be assumed that all His residues are involved in the coordination of the Zn(II) ion directly or indirectly through the steric effect. The L3 ligand has the best affinity to metal ions already in an acidic environment and throughout the range of the tested pH. The L2 ligand containing the same albumin-like motif as L3 starts to bind metal ions identically as L3 at lower pH values but with significantly less affinity. It could be concluded that although L1 and L2 have the same number of histidyl residues, their mutual position affects the effectiveness of metal ion binding at a given pH value. Above pH 6.5, L1 binds the Zn(II) with higher stability than that observed for L2. The nearly identical amount of metal ions bound might suggest that identical donor atom sets for both systems Zn(II)/L1 and Zn(II)/L2 are involved in Zn(II) coordination.

Comparison of the Zn(II) binding of the tested ligands L1, L2, and L3 with their shorter 12-mer counterparts shows (Figure 3B–D) that in all cases, the longer fragments show a better affinity for metal ions. This may confirm the previously proposed positive effect of elongation and the presence of an additional His residue in the sequences of the tested ligands.

Lipophilicity seems to be one of the most important physicochemical properties of biologically active compounds influencing their biological activity, related to the ADME (Absorption, Distribution, Metabolism, and Excretion) profile for the tested substance. This is due to the better affinity of a given chemical compound with biological membranes, better permeability, and thus better access to the site of action in the body.

Due to the hydrophobic nature of some amino acid residues, as well as the net value of the peptide, the lipophilicity of CAMPs seems to be a feature that should be investigated.

As we suggested previously [21], lipophilicity may also be a factor related to the antimicrobial activity of peptide ligands. The partition coefficient logP_o/w_ values for studied peptides and their metal complexes were determined experimentally based on the shake-flask method [32] for all studied ligands and their metal complexes, and the results are shown in Table 4.

Since L1, L2, and L3 ligands as well as their copper complexes are soluble in water, they revealed hydrophilic character. Zinc complexes were not soluble above pH 9 and 7 for L1 and L2, L3, respectively. It was surprising that upon contact with the inorganic n-octanol phase, Zn(II)/L1 complexes formed in the water were precipitated, which prevented further measurement of the partition coefficient logP_o/w_. This undoubtedly proves the hydrophilic nature of Zn (II)/L1 complexes. In the case of zinc complexes of the remaining ligands, no precipitate was observed, so experimental values of logP_o/w_ were determined. In comparison to free ligands, the lipophilicity of Cu(II) complexes is lower for all studies’ systems. For L2 and L3, and their Zn(II) complex forms, we observed a slight difference in the partition coefficient concerning the ligands themselves, a slight decrease in lipophilicity in the case of Zn(II)/L2 complexes, and an increase in the case of Zn(II)/L3. Increasing the lipophilicity of complex compounds concerning the free ligands translates into their better antimicrobial properties, which is confirmed by the results of biological tests.

The obtained results are in good agreement with results for 12-mer ligands [21]. Compared to shorter analogs, the 15-mer peptides show greater lipophilic properties, as indicated by the higher values of the partition coefficient. Copper complexes are less lipophilic only in the case of Cu(II)/L1 (logP_o/w_ = −0.96, while for 12-mer logP_o/w_ = −0.62 [21]). It is also worth noting that for L3, the Zn(II)/L3 complexes show much better lipophilic properties than the copper complexes, as well as the pure L3 ligand.

In Tables S6 and S7 the measured MIC values for selected antibiotic and AMP compounds as well as its complexes with metal ions are presented [33,34,35,36]. MIC for studied systems are higher in comparison to the selected antibiotics and AMP compounds except *S. epidermidis*.

## 3. Materials and Methods

All peptides (KSAFELPHYGLLAHQ (L1), KSHFELPAYGLLAHQ (L2), and KSHFELPHYGLLAHQ (L3)) were commercial products purchased from Zhejiang Ontores Biotechnologies Co., Ltd., Hangzhou, China, (certified purity: 98%) and were used without further purification. The molecular weight of each compound was checked by ESI-MS. The exact concentration of 0.1 M KOH solution (Merck, Darmstadt, Germany) was standardized potentiometrically using potassium hydrogen phthalate.

The complex formation equilibria with metal ions were studied by potentiometry and mass spectrometry, and for Cu(II) additionally by UV-Vis and CD spectroscopy. Zinc hydrolysis precipitation was not observed in the measured pH range at 1 mM concentration of L1 and L2, and ratio of metal ion to ligand 0.5:1, 1:1.1.

### 3.1. Biological Studies

#### In Vitro Antimicrobial Activity

The antimicrobial properties of three peptides (L1, L2, L3) and metal(II) complexes (molar ratio, 1:1) were tested on human pathogenic strains. *E. coli ATCC 25922*, *E. faecalis ATCC 29212*, *P. aeruginosa ATCC 15422*, and *S. epidermidis KCTC 1917* were grown at 37 °C in Luria Bertani Broth (LB; 10 g/L bacto-tryptone, 5 g/L bacto-yeast extract, 10 g/L NaCl). The *C. albicans ATCC 20231* was grown in a 6.7 g/L yeast nitrogen base (YNB) broth (Difco Laboratories) containing 2% D-glucose at 37 °C.

The minimum inhibitory concentration (MIC) values of the peptides and their metal(II) complexes were determined using the microdilution broth method in 96-well microplates (Sarstedt, Nümbrecht, Germany) [37]. The serial dilutions of tested compounds (0–500 µM) were dissolved in LB medium for bacteria and YNB medium for C. albicans. The inoculum (2.5 µL) of each strain (108 CFU mL^−1^) was placed into each well containing 200 µL of serial dilutions of the tested peptides and their metal(II) complexes. Negative and growth control wells did not contain the tested compounds. Bacteria and C. albicans incubated with metal ions were used as additional controls. After 24 h of incubation at 37 °C, the optical density at 600 nm of each well was measured using a multifunctional microplate reader (Synergy™ H1, BioTek, Winooski, VT, USA). The MIC was defined as the lowest concentration of the tested compounds that completely inhibited visible bacterial and *C. albicans* growth.

### 3.2. Potentiometric Studies

The titrations were performed using a Metrohm 809 Titrando (Herisau, Switzerland) system equipped in combined glass electrode (Metrohm 6.0224.100) which was calibrated daily in hydrogen concentrations using HCl (Merck, Darmstadt, Germany) (~0.004M) according to the procedure of Irving et al. [38]. Alkali, CO_2_-free 0.1017 M KOH solution (POCh, Gliwice, Poland) was added from a Metrohm 800 Dosino auto burette. The ionic strength was fixed at I = 0.1 M with KCl (POCh). The purity and exact concentrations of the ligands were determined by the method of Gran [39]. The stock solutions of metal ions were prepared by dissolving an appropriate amount of chlorides in an aqueous HCl solution. All the titrations were carried out on 2.0 mL samples in a thermostatted cell at 25 ± 0.2 °C under a stream of Ar. SUPERQUAD (Leeds, England) computer program that uses non-linear least-squares methods [40] and HYPERQUAD2008 (Leeds, England) were applied to calculate the stability constants. The results were obtained in the form of concentration overall stability constants β_pqr_= [M_p_H_q_L_r_]/[M]_p_[H]_q_[L]_r_, where M stands for metal. H is proton and L is the deprotonated form of the ligand. Triplicate titrations of the free ligand and the complexes were carried out at metal to ligand ratios 1:1.1 and 1:2. The ligand concentration was 1.0 × 10^−3^ M in all titrations. The distribution curves of the protonated species of ligands as a function of pH were calculated using the HySS2009 (Leeds, England) program [41].

### 3.3. Mass Spectrometry Measurements

All ESI-MS experiments were performed on a compact mass spectrometer (Bruker Daltonics, Bremen, Germany) equipped with a standard ESI source. The instruments were operated in the positive-ion mode and calibrated with the Tunemix mixture (Agilent Technologies, Palo Alto, CA, USA) in a quadratic method. Spectra were recorded for samples dissolved in H_2_O with an M(II):L molar ratio of 1:0.5, 1:1.1, and 1:2. pH was adjusted by adding NaOH or HCOOH and checked by a Metrohm 913 pH-meter (Herisau, Switzerland). Analyte solutions (150 μL) were introduced at a flow rate of 180 μL/h. The instrument parameters were as follows: scan range: 50–3000 m/z, drying gas: nitrogen, flow rate: 4.0 L/min, temperature: 200 °C, and potential between the spray needle and the orifice: 4.0 kV.

For MS spectra analysis, Bruker Compass Data Analysis 4.2 software was used. A sophisticated numerical annotation procedure (SNAP) algorithm was used for finding peaks. The relative intensities (Tables S1–S4, Supplementary Materials) were read directly from the MS spectra in the Data Analysis 4.2 program. Figures S1–S9 contain raw MS spectra with intensities on the y-axis and isotopic pattern enlargements for the main species present.

### 3.4. Spectroscopic Studies

The absorption spectra were recorded on a Varian Cary50 spectrophotometer (part of Agilent Technologies, Mulgrave, Victoria, Australia), in the range 200–800 nm, using a quartz cuvette with an optical path of 1 cm. Circular dichroism (CD) spectra were recorded on a Jasco J-1500 CD spectrometer (Tokyo, Japan) in the 200–800 nm range, using a quartz cuvette with an optical path of 1 cm in the visible and near-UV range or with a cuvette with an optical path of 0.01 cm in the wavelength range 180–300 nm. The Cu(II)/peptide complexes were prepared in a water solution of HCl at I = 0.1 M (KCl). The concentrations of solutions used for spectroscopic studies were similar to those employed in the potentiometric experiment. The metal to ligand ratio was 1:1.1. pH was adjusted with appropriate amounts of HCl or NaOH. UV-Vis and CD spectroscopic parameters of complexes were calculated from the spectra at the pH values corresponding to the maximum concentration of each particular species, based on distribution diagrams.

### 3.5. Lipophilicity Measurements

The shake-flask method was used to experimentally determine the value of logP_o/w_ for all three ligands (L1, L2, and L3) and their metal (Cu(II) and Zn(II)) complexes (M(II)/L1, M(II)/L2, M(II)/L3). All experiments were run in aqueous (50 mM HEPES, pH 7.4 and *I*_KCl_ = 0.16 M, 25 °C) and 1-octanol solvents; 0.75 mL of 1-octanol and 0.75 mL of 1 mM solution of the studied ligand or complex (in the molar ratio 1:1 [L] = 0.5 mM) were mixed. The samples were vortexed (~5 min), shaken manually (~2 min), and centrifuged (~3 min, 6000 rpm). The two layers were carefully separated and measured by UV-Vis spectroscopy, applying the Beer–Lambert law to determine the ligand and complex concentration in the analyzed layer (*ε* and *λ*_max_ are given in Table S5). Measurements were made in triplicate. For Zn(II), precipitations of the complex in the organic phase were observed for all studied ligands in used concentrations.

### 3.6. Computational Study

The analysis of conformational space of the L1 and its CuL complexes was analyzed using the PM6 method, taking into account solvent effects (PCM model), which was quite a successful approach in determining structures of bioinorganic complexes with transition metals [42,43,44]. All the calculations were performed within unrestricted formalism using the Gaussian 09 suite of programs [45].

## 4. Conclusions

Antimicrobial peptides could undoubtedly be very effective agents against pathogens, especially in combination with metal ions, such as Cu(II) and Zn(II). In the present work, the thermodynamic and biological properties of the 15-amino acid fragment of the mucin 7 protein (L3) and two of its analogs differing in the location of histidyl residues were investigated (L1 and L2). The work refers to the study of a shorter, 12-meter fragment, showing antimicrobial properties against Gram-positive bacteria [21]. Based on the obtained results, it was concluded that the original fragment containing three His residues in its sequence (L3) shows lower stability in the forms composed of copper ions and higher with zinc ions in comparison to the di-histidyl analogs. This is most likely related to the steric effects taking place between the coordination sphere of the complex and the side chains of amino acid residues in the ligand peptide sequences.

All studied ligands and their metal complexes were screened for both antibacterial and antifungal activities in vitro against *E. faecalis*, *S. epidermidis*, *E. coli*, *P. aeruginosa*, and *C. albicans*, revealing activity towards all tested pathogens, except for L3, which showed no activity against *E. coli* and *C. albicans*. In fact, we have proved that 15-mer MUC7 peptides and their metal complexes can display significant antimicrobial activity, which highlights the potential interest of the peptides presented in this work. Moreover, the binding of the metal may favor interaction with the bacterial cell wall and the components of the membrane, resulting in potentiation of antibacterial activity; however, additional experiments need to be performed to confirm this hypothesis.

## Figures and Tables

**Table 1:** Antimicrobial activity of KSAFELPHYGLLAHQ (L1), KSHFELPAYGLLAHQ (L2), KSHFELPHYGLLAHQ (L3), and their metal(II) complexes against pathogenic microorganisms. Values are the average of three independent experiments performed in three wells.

Ligands/Complexes MIC (µM)	*E. faecalis* ATCC 29212	*S. epidermidis* KCTC 1917	*E. coli* ATCC 25922	*P. aeruginosa* ATCC 15422	*C. albicans* ATCC 10231
L1	145 ± 3.1	132 ± 1.6	412 ± 2.4	285 ± 2.6	436 ± 2.4
L1/Cu(II)	132 ± 1.4	105 ± 2.6	388 ± 1.9	254 ± 1.3	412 ± 3.5
L1/Zn(II)	108 ± 1.3	85 ± 1.5	356 ± 2.6	230 ± 3.1	377 ± 2.7
L2	163 ± 2.2	147 ± 1.6	416 ± 0.5	302 ± 2.8	469 ± 1.4
L2/Cu(II)	134 ± 1.4	133 ± 1.4	401 ± 1.4	288 ± 3.2	455 ± 1.9
L2/Zn(II)	125 ± 1.4	114 ± 2.2	389 ± 2.4	281 ± 0.9	414 ± 2.6
L3	241 ± 1.6	210 ± 2.6	>500	313 ± 2.5	>500
L3/Cu(II)	224 ± 3.1	194 ± 2.1	>500	294 ± 1.3	>500
L3/Zn(II)	198 ± 2.8	192 ± 0.3	>500	277 ± 1.8	>500

**Table 2:** Potentiometric data for the proton, M(II) (Cu(II) and Zn(II)) KSAFELPHYGLLAHQ (L1), KSHFELPAYGLLAHQ (L2), and KSHFELPHYGLLAHQ (L3). M(II) to peptide molar ratio, 1:1.1, T:298 K, I = 0.1 M, [M(II)] = [L1–3] = 0.0005 M.

	L1		L2		L3	
Species	logβ	pK	logβ	pK	logβ	pK
HL	9.92 (2)	9.92	10.50 (2)	10.50	10.01 (2)	10.01
H_2_L	19.36 (2)	9.44	20.08 (2)	9.58	19.36 (2)	9.35
H_3_L	26.95 (2)	7.59	27.50 (3)	7.42	26.72 (3)	7.36
H_4_L	33.75 (3)	6.62	34.22 (3)	6.72	33.47 (3)	6.75
H_5_L	39.73 (4)	5.98	39.84 (4)	5.62	39.81 (4)	6.34
H_6_L	43.74 (3)	4.01	43.44 (4)	3.60	45.21 (4)	5.40
H_7_L	46.40 (2)	2.66	45.79 (2)	2.35	48.87 (2)	3.66
H_8_L					51.16 (3)	2.29
CuH_5_L					43.92 (6)	
CuH_3_L	33.16 (4)		34.18 (1)		36.09 (1)	7.83 (2H+)
CuH_2_L	27.90 (2)	5.26	30.37 (2)	3.81	31.25 (3)	4.84
CuHL	19.09 (1)	8.81	25.98 (1)	4.39	24.86 (4)	6.39
CuL	10.20 (2)	8.89	19.52 (3)	6.46	17.81 (5)	7.05
CuH_−1_L	0.59 (1)	9.61	10.09 (5)	9.43	8.19 (7)	9.62
CuH_−2_L	−10.27 (1)	10.86	0.03 (6)	10.06	−1.66 (3)	9.85
ZnH_4_L					37.44 (5)	
ZnH_3_L			31.31 (7)		31.72 (2)	5.72
ZnH_2_L	24.47 (10)					
ZnHL	16.49 (4)	7.98	16.97 (2)	14.32 (2H+)	18.11 (2)	13.60 (2H+)
ZnL	7.78 (4)	8.72				

**Table 3:** Spectroscopic parameters of Cu(II) complexes of KSAFELPHYGLLAHQ (L1), KSHFELPAYGLLAHQ (L2), and KSHFELPHYGLLAHQ (L3). M(II) to peptide molar ratio, 1:1, Table 298. K, I = 0.1 M, [M(II)] = [L1−3] = 0.0005 M.

Species	L1				L2				L3			
	UV-Vis		CD		UV-Vis		CD		UV-Vis		CD	
	λ nm	ε M^−1^ cm^−1^	λ nm	Δε M^−1^ cm^−1^	λ nm	ε M^−1^ cm^−1^	λ nm	Δε M^−1^ cm^−1^	λ nm	ε M^−1^ cm^−1^	λ nm	Δε M^−1^ cm^−1^
CuH_5_L									mix		mix	
CuH_3_L	mix	mix	mix	mix	mix	mix	mix	mix	mix		mix	
CuH_2_L	612	34	230	9.233	mix	mix	mix	mix	522	108	252	0.184
			250	−1.340							274	−0.880
			317	0.354							312	0.648
			690	−0.292							489	0.293
											567	−0.304
CuHL	mix	mix	mix	mix	518	82	228 251 275 312 488 567	−9.070 0.512 −2.736 1.346 0.668 −0.588	520	120	252 273 312 486 563	0.515 −2.930 1.460 0.693 −0.681
CuL	522	141	235 250 275 311 532	−1.765 1.067 −2.062 0.801 −0.807	518	160	229 250 276 311 488 571	−8.143 1.675 −2.134 1.461 0.796 −0.507	520	137	254 274 312 486 562	−0.727 −2.730 1.420 0.684 −0.713
CuH_−1_L	520	140	232 248 274 309 532	−4.055 1.791 −2.660 1.012 −1.361	519	167	228 251 276 311 489 568	−9.531 1.746 −2.124 1.624 0.637 −0.526	mix		mix	
CuH_−2_L	520	157	235 250 275 311 532	−0.883 0.534 −1.032 0.400 −0.403	519	167	227 250 277 311 490 561	−10.45 1.868 −2.868 1.649 0.562 −0.609	520	160	253 274 311 486 567	2.387 −2.947 1.371 0.766 −0.713

**Table 4:** Experimentally determined log P_o/w_ values. Shake-flask method; P_o/w_ = []_1-oktanol_/[]_HEPES_; pH 7.2; T = 25 °C; concentration determined by UV-Vis absorption. Experimentally measured molar extinction coefficients (ε) and absorbance maxima (λmax) for L1, L2 and L3 are collected in Table S5.

logP_o/w_			
Species	L1	L2	L3
L	−0.41 ± 0.3	−0.56 ± 0.3	−0.98 ± 0.3
Cu(II)/L	−0.96 ± 0.3	−1.33 ± 0.3	−1.44 ± 0.3
Zn(II)/L	------	−0.96 ± 0.3	−0.57 ± 0.3

## Data Availability

Not applicable.

## Supplementary Materials

The following supporting information can be downloaded at: https://www.mdpi.com/article/10.3390/ijms23010418/s1.
