# Supplementary material for: The Potential Antimicrobial Action of Human Mucin 7 15-Mer Peptide and Its Metal Complexes
Source: Int J Mol Sci. 2021 Dec 30;23(1):418. doi: 10.3390/ijms23010418 (PMC8745124; doi:10.3390/ijms23010418)
Supplement: Supplementary file 1 [file ijms-23-00418-s001.zip › ijms-1501899-supplementary.pdf]

Table S1. ESI-MS data for Cu(II) complexes with L1. Metal: peptide ratio: 0.5:1, 1:1 and 1.5:1.

complex	found [a]	calc.[b]	err [ppm]	relative intensity [%]
L1 : Cu (1:0.5) pH 0.5				
$[L+Cu]^{2+}$	886.4124	886.4087	4.17	22
$[L+Cu+Na-H]^{2+}$	897.4029	897.3997	3.57	6
$[L+Cu+H]^{3+}$	591.2761	591.2749	2.03	13
$[2L+Cu+H]^{3+}$	1161.241	1161.2379	2.67	1
L1 : Cu (1:0.5) pH 7.2				
$[L+Cu+Na-H]^{2+}$	897.4042	897.3997	5.01	92
$[L+Cu+2Na-2H]^{2+}$	908.3951	908.3907	4.84	100
$[L+Cu+3Na-3H]^{2+}$	919.3851	919.3816	3.81	43
$[2L+Cu+2Na-H]^{3+}$	1175.8964	1175.8925	3.32	2
$[2L+Cu+3Na-2H]^{3+}$	1183.2247	1183.2198	4.14	2
L1 : Cu (1:0.5) pH 9.2				
$[L+Cu+2Na-2H]^{2+}$	908.3964	908.3907	6.27	100
$[L+Cu+3Na-3H]^{2+}$	919.3861	919.3816	5.78	77
$[L+Cu+4Na-4H]^{2+}$	930.377	930.3726	4.73	33
$[2L+Cu+4Na-3H]^{3+}$	1190.5512	1190.5471	3.44	2
L1 : Cu (1:1) pH 4.3				
$[L+Cu+Na-H]^{2+}$	897.4042	897.3997	5.01	92
$[L+Cu+2Na-2H]^{2+}$	908.3951	908.3907	4.84	100
$[L+Cu+3Na-3H]^{2+}$	919.3851	919.3816	3.81	43
[2L+Cu+2Na-H]3+	1175.8964	1175.8925	3.32	2
[2L+Cu+3Na-2H]3+	1183.2247	1183.2198	4.14	2
L1 : Cu (1:1) pH 7.3				
$[L+Cu+Na-H]^{2+}$	897.4054	897.3997	6.35	65
$[L+Cu+2Na-2H]^{2+}$	908.3962	908.3907	6.05	100
$[L+Cu+3Na-3H]^{2+}$	919.3867	919.3816	5.55	58
L1 : Cu (1:1) pH 9.2				
$[L+Cu+2Na-2H]^{2+}$	908.3988	908.3907	8.92	86
$[L+Cu+3Na-3H]^{2+}$	919.3895	919.3816	8.59	100
$[L+Cu+4Na-4H]^{2+}$	930.3805	930.3726	8.49	82

L1 : Cu (1:1.5) pH 4.3				
$[L+Cu+Na-H]^{2+}$	897.4105	897.3997	12.03	16
$[L+Cu+2Na-2H]^{2+}$	908.403	908.3907	13.54	12
$[L+Cu+3Na-3H]^{2+}$	919.3889	919.3816	7.94	5
L1 : Cu (1:1.5) pH 7.3				
$[L+Cu+Na-H]^{2+}$	897.4093	897.3997	10.70	57
$[L+Cu+2Na-2H]^{2+}$	908.4000	908.3907	10.24	100
$[L+Cu+3Na-3H]^{2+}$	919.3909	919.3816	10.12	74
L1 : Cu (1:1.5) pH 9.3				
$[L+Cu+2Na-2H]^{2+}$	908.4018	908.3907	12.22	68
$[L+Cu+3Na-3H]^{2+}$	919.3928	919.3816	12.18	63
$[L+Cu+4Na-4H]^{2+}$	930.3829	930.3726	11.07	45
$[L+2Cu+2Na-4H]^{2+}$	938.8626	938.8476	15.98	81
$[L+2Cu+3Na-4H]^{3+}$	633.5700	633.5615	13.42	21
$[L+2Cu+4Na-5H]^{3+}$	640.8962	640.8888	11.55	20
$[L+2Cu+5Na-6H]^{3+}$	648.2265	648.2161	16.04	20

[a] monoisotopic mass found experimentally on a compact™ mass spectrometer (Bruker Daltonics. Bremen. Germany)

[b] monoisotopic mass of the indicated ion formed by the ligand calculated by Compass DataAnalysis 4.2

Table S2. ESI-MS data for Cu(II) complexes with L2. Metal: peptide ratio: 0.5:1, 1:1 and 1.5:1.

complex	found [a]	calc.[b]	err [ppm]	relative intensity [%]
L2 : Cu (1:0.5) pH 4.3				
$[L+Cu]^{2+}$	886.4091	886.4087	0.45	17
$[L+Cu+Na-H]^{2+}$	897.3994	897.3997	-0.33	5
$[L+Cu+H]^{3+}$	591.2748	591.2749	-0.17	3
$[2L+Cu+H]^{3+}$	1161.2356	1161.2379	-1.98	2
L2 : Cu (1:0.5) pH 7.4				
$[L+Cu+Na-H]^{2+}$	897.4039	897.3997	4.68	22
$[L+Cu+2Na-2H]^{2+}$	908.3929	908.3907	2.42	35
$[L+Cu+3Na-3H]^{2+}$	919.3836	919.3816	2.18	29
$[2L+Cu+2Na-H]^{3+}$	1175.8934	1175.8925	0.77	1
$[2L+Cu+3Na-2H]^{3+}$	1183.2204	1183.2198	0.51	3
$[2L+Cu+4Na-3H]^{3+}$	1190.5477	1190.5471	0.50	2
L2 : Cu (1:0.5) pH 9.5				
$[L+Cu+2Na-2H]^{2+}$	908.3932	908.3907	2.75	20
$[L+Cu+3Na-3H]^{2+}$	919.3808	919.3816	-0.87	34
$[L+Cu+4Na-4H]^{2+}$	930.3715	930.3726	-1.18	27
$[2L+Cu+4Na-3H]^{3+}$	1190.5459	1190.5471	-1.01	2
$[2L+Cu+5Na-4H]^{3+}$	1197.9716	1197.9745	-2.42	2
$[2L+Cu+6Na-5H]^{3+}$	1205.1977	1205.2018	-3.40	2
L2 : Cu (1:1) pH 4.4				
$[L+Cu]^{2+}$	886.4066	886.4087	-2.37	76
$[L+Cu+Na-H]^{2+}$	897.3975	897.3997	-2.45	100
$[L+Cu+2Na-2H]^{2+}$	908.3878	908.3907	-3.19	49
L2 : Cu (1:1) pH 7.4				
$[L+Cu+3Na-2H]^{3+}$	613.2557	613.2568	-1.79	100
$[L+Cu+4Na-3H]^{3+}$	620.5828	620.5842	-2.26	89
$[L+Cu+5Na-4H]^{3+}$	627.9098	627.9115	-2.71	40
L2 : Cu (1:1) pH 9.4				
$[L+Cu+3Na-2H]^{3+}$	613.2563	613.2568	-0.82	30
$[L+Cu+4Na-3H]^{3+}$	620.5842	620.5842	0.00	100
$[L+Cu+5Na-4H]^{3+}$	627.9110	627.9115	-0.80	40

$[L+2Cu+4Na-5H]^{3+}$	640.8886	640.8888	-0.31	20
$[L+2Cu+5Na-6H]^{3+}$	648.2170	648.2161	1.39	9
L2 : Cu (1:1.5) pH 4.4				
[L+Cu+2Na-H]3+	605.929	605.9295	-0.83	100
$[L+Cu+3Na-2H]^{3+}$	613.2560	613.2568	-1.30	80
$[L+Cu+4Na-3H]^{3+}$	620.5826	620.5842	-2.58	33
$[L+2Cu+4Na-5H]^{3+}$	640.8891	640.8888	0.47	3
$[L+2Cu+5Na-6H]^{3+}$	648.2217	648.2161	8.64	3
L2 : Cu (1:1.5) pH 7.5				
$[L+Cu+3Na-2H]^{3+}$	613.2576	613.2568	1.30	84
$[L+Cu+4Na-3H]^{3+}$	620.5848	620.5842	0.97	100
$[L+Cu+5Na-4H]^{3+}$	627.9121	627.9115	0.96	65
$[L+2Cu+4Na-5H]^{3+}$	640.8904	640.8888	2.50	10
$[L+2Cu+5Na-6H]^{3+}$	648.2221	648.2161	9.26	6
L2 : Cu (1:1.5) pH 9.5				
$[L+Cu+4Na-3H]^{3+}$	620.5854	620.5842	1.93	60
$[L+Cu+5Na-4H]^{3+}$	627.9126	627.9115	1.75	43
$[L+Cu+6Na-5H]^{3+}$	635.2396	635.2388	1.26	20
$[L+2Cu+4Na-5H]^{3+}$	640.8904	640.8888	2.50	75
$[L+2Cu+5Na-6H]^{3+}$	648.2177	648.2161	2.47	60
$[L+2Cu+6Na-7H]^{3+}$	655.5451	655.5434	2.59	25

[a] monoisotopic mass found experimentally on a compact™ mass spectrometer (Bruker Daltonics. Bremen. Germany)
[b] monoisotopic mass of the indicated ion formed by the ligand calculated by Compass DataAnalysis 4.2

Table S3. ESI-MS data for Cu(II) complexes with L3. Metal: peptide ratio: 0.5:1, 1:1 and 1.5:1

L3: Cu (1:0.5) pH 4.3				
complex	found [a]	calc.[b]	err [ppm]	relative intensity [%]
$[L+Cu]^{2+}$	919.4216	919.4196	2.18	19
$[L+Cu+Na-H]^{2+}$	930.4126	930.4106	2.15	4
$[L+Cu+H]^{3+}$	613.282	613.2822	-0.33	15
$[2L+Cu+H]^{3+}$	1205.2527	1205.2524	0.25	2
L3: Cu (1:0.5) pH 7.5				
$[L+Cu+2Na-H]^{3+}$	627.9388	627.9368	3.19	10
$[L+Cu+3Na-2H]^{3+}$	635.2652	635.2641	1.73	33
$[L+Cu+4Na-3H]^{3+}$	642.5923	642.5914	1.40	28
$[2L+Cu+2Na-H]^{3+}$	1219.9133	1219.907	5.16	1
$[2L+Cu+3Na-2H]^{3+}$	1227.2387	1227.2344	3.50	2
$[2L+Cu+4Na-3H]^{3+}$	1234.5653	1234.5617	2.92	2
$[2L+Cu+5Na-4H]^{3+}$	1241.8904	1241.889	1.13	2
L3 : Cu (1:0.5) pH 9.5				
$[L+Cu+4Na-4H]^{2+}$	963.3877	963.3835	4.36	14
$[L+Cu+3Na-2H]^{3+}$	635.2673	635.2641	5.04	10
$[L+Cu+4Na-3H]^{3+}$	642.5929	642.5914	2.33	31
$[2L+Cu+4Na-3H]^{3+}$	1234.5691	1234.5617	5.99	1
$[2L+Cu+5Na-4H]^{3+}$	1241.8947	1241.889	4.59	2
L3 : Cu (1:1) pH 4.3				
$[L+Cu]^{2+}$	919.4237	919.4196	4.46	27
$[L+Cu+H]^{3+}$	613.2842	613.2822	3.26	18
$[L+Cu+Na]^{3+}$	620.6107	620.6095	1.93	29
$[2L+Cu+H]^{3+}$	1205.2557	1205.2524	2.74	1
L3 : Cu (1:1) pH 7.3				
$[L+Cu+3Na-2H]^{3+}$	635.2661	635.2641	3.15	97
$[L+Cu+4Na-3H]^{3+}$	642.5934	642.5914	3.11	72
$[L+Cu+5Na-4H]^{3+}$	649.9205	649.9187	2.77	33
L3 : Cu (1:1) pH 9.3				
$[L+Cu+3Na-2H]^{3+}$	635.2647	635.2641	0.94	44

$[L+Cu+4Na-3H]^{3+}$	642.5918	642.5914	0.62	100
$[L+Cu+5Na-4H]^{3+}$	649.9189	649.9187	0.31	44
L3 : Cu (1:1.5) pH 4.4				
$[L+Cu+Na-H]^{2+}$	930.4151	930.4106	4.84	53
$[L+Cu+Na]^{3+}$	620.6119	620.6095	3.87	81
$[L+Cu+2Na-H]^{3+}$	627.9391	627.9368	3.66	98
$[2L+Cu+H]^{3+}$	1205.261	1205.2524	7.14	1
L3 : Cu (1:1.5) pH 7.2				
$[L+Cu+3Na-2H]^{3+}$	635.2666	635.2641	3.94	100
$[L+Cu+4Na-3H]^{3+}$	642.5938	642.5914	3.73	72
$[L+Cu+5Na-4H]^{3+}$	649.921	649.9187	3.54	48
L3 : Cu (1:1.5) pH 9.3				
$[L+Cu+4Na-4H]^{2+}$	963.3899	963.3835	6.64	33
$[L+Cu+4Na-3H]^{3+}$	642.5955	642.5914	6.38	98
$[L+Cu+5Na-4H]^{3+}$	649.9229	649.9187	6.46	58

[a] monoisotopic mass found experimentally on a compact™ mass spectrometer (Bruker Daltonics. Bremen. Germany)

[b] monoisotopic mass of the indicated ion formed by the ligand calculated by Compass DataAnalysis 4.2

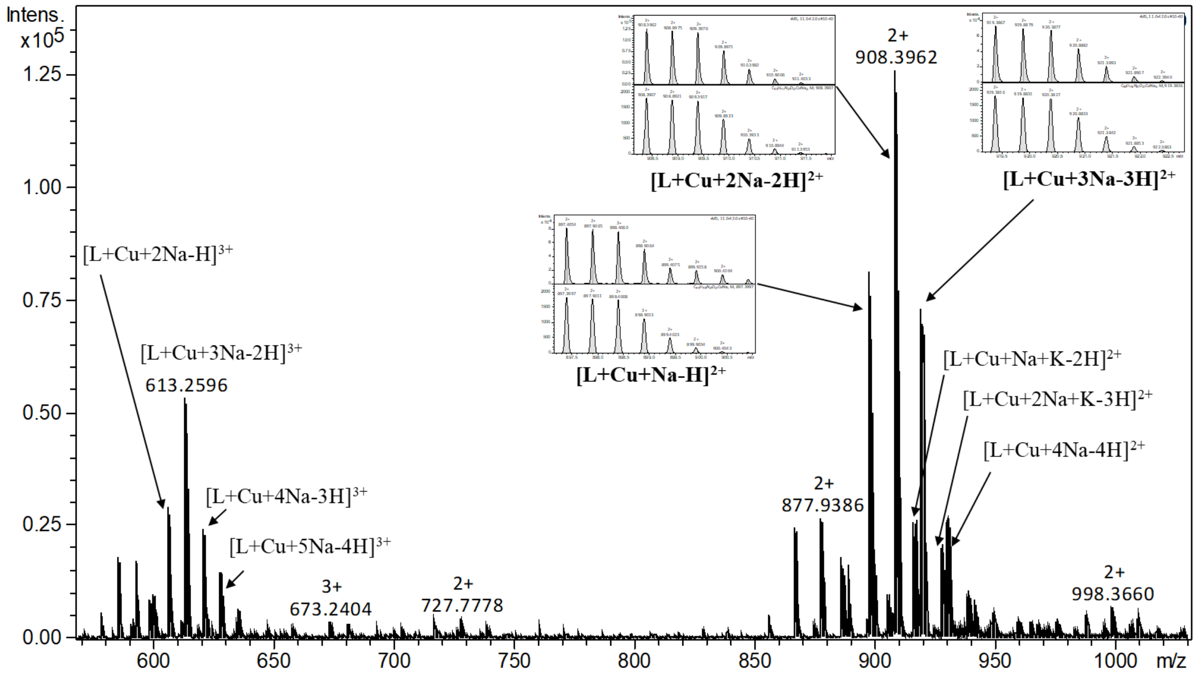

Figure S1. ESI-MS data for Cu(II) complexes of L1. Enlarged pictures: experimental (higher panel) *vs* simulated (lower panel) isotopic pattern of the complexes; shown only for main species. L1 : Cu(II) (1:1) pH 7.3.

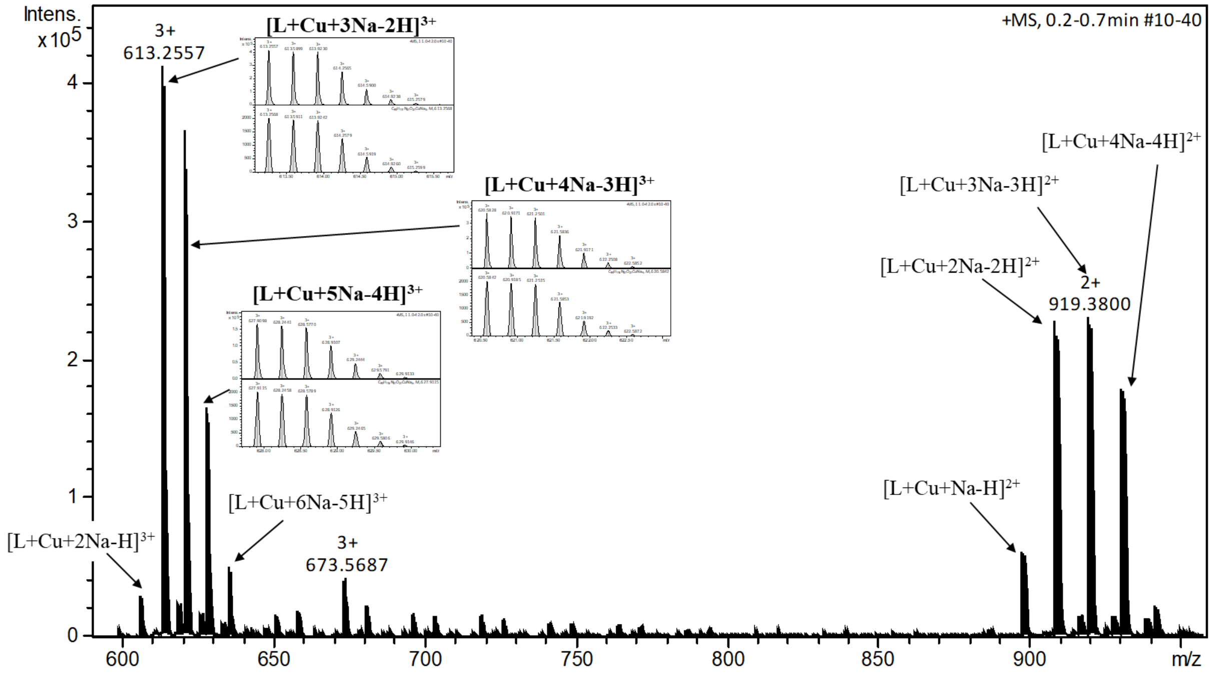

Figure S2. ESI-MS data for Cu(II) complexes of L2. Enlarged pictures: experimental (higher panel) *vs* simulated (lower panel) isotopic pattern of the complexes; shown only for main species. L2 : Cu(II) (1:1) pH 7.4.

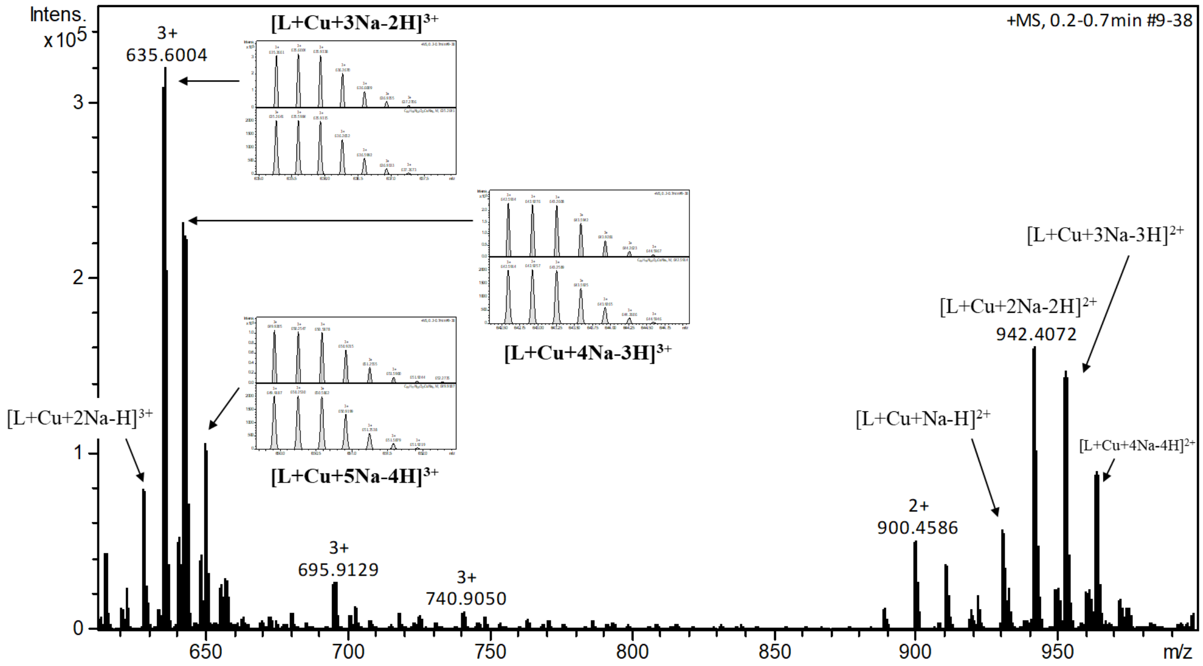

Figure S3. ESI-MS data for Cu(II) complexes of L3. Enlarged pictures: experimental (higher panel) *vs* simulated (lower panel) isotopic pattern of the complexes; shown only for main species. L3 : Cu(II) (1:1) pH 7.3.

Table S4. ESI-MS data for Zn(II) complexes with L3. Metal: peptide ratio: 1:1.

complex	found [a]	calc.[b]	err [ppm]	relative intensity [%]
L1 : Zn (1:1) pH 7.2				
$[L+K+H]^{2+}$	874.9245	874.9297	-5.94	100
$[L+Zn+K+4Na-5H]^{2+}$	949.8473	949.8503	-3.16	1
$[L+Zn+2K+4Na-6H]^{2+}$	968.8237	968.8283	-4.75	2
L1 : Zn (1:1) pH 9.0				
[L+3K-H]2+	912.8792	912.8856	-7.01	100
$[L+Zn+K+4Na-5H]^{2+}$	949.8487	949.8503	-1.68	4
$[L+Zn+2K+4Na-6H]^{2+}$	968.8230	968.8283	-5.47	12
$[L+Zn+2K+10Na-12H]^{2+}$	1034.7699	1034.7741	-4.06	3
$[L+Zn+3K+9Na-12H]^{2+}$	1042.757	1042.7611	-3.93	3
L2 : Zn (1:1) pH 7.2				
$[L+2H]^{2+}$	855.9447	855.9517	-8.18	100
L2 : Zn (1:1) pH 8.9				
$[L+2H]^{2+}$	893.9005	893.9076	-7.94	100

$[L+Zn+K+5Na-6H]^{2+}$	960.8349	960.8413	-6.66	6
$[L+Zn+2K+4Na-6H]^{2+}$	968.8216	968.8283	-6.92	3
$[L+Zn+2K+10Na-12H]^{2+}$	1035.2692	1035.2755	-6.09	1
L3: Zn (1:1) pH 7.2				
$[L+K+H]^{2+}$	908.4348	908.442	-7.93	100
$[L+Zn+K+5Na-6H]^{2+}$	993.8455	993.8522	-6.74	0.7
$[L+Zn+2K+4Na-6H]^{2+}$	1001.8350	1001.8392	-4.19	0.25
L3: Zn (1:1) pH 9.0				
$[L+2K]^{2+}$	926.9101	926.9185	-9.06	100
$[L+Zn+K+4Na-5H]^{2+}$	982.8548	982.8612	-6.51	2
$[L+Zn+K+5Na-6H]^{2+}$	993.8443	993.8522	-7.95	4
$[L+Zn+2K+4Na-6H]^{2+}$	1001.8308	1001.8392	-8.38	3

[a] monoisotopic mass found experimentally on a compact™ mass spectrometer (Bruker Daltonics. Bremen. Germany)
[b] monoisotopic mass of the indicated ion formed by the ligand calculated by Compass DataAnalysis 4.2

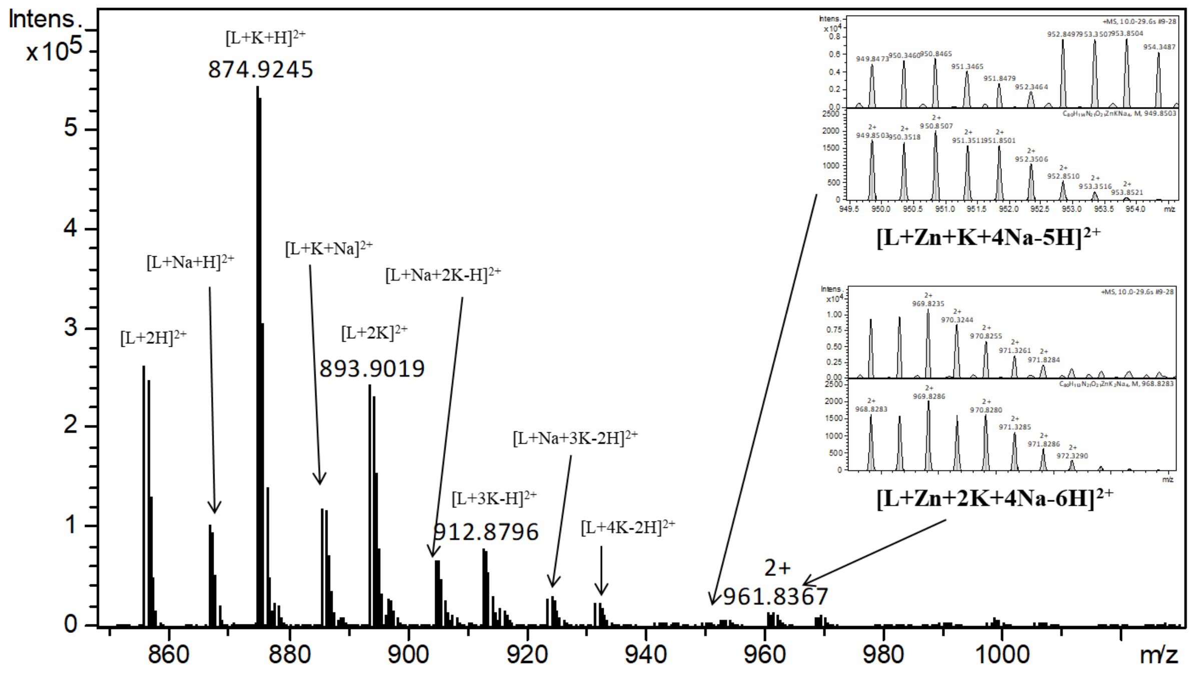

Figure S4. ESI-MS data for Zn(II) complexes of L1. Enlarged pictures: experimental (higher panel) *vs* simulated (lower panel) isotopic pattern of the complexes; shown only for main species. L1 : Zn(II) (1:1) pH 7.2.

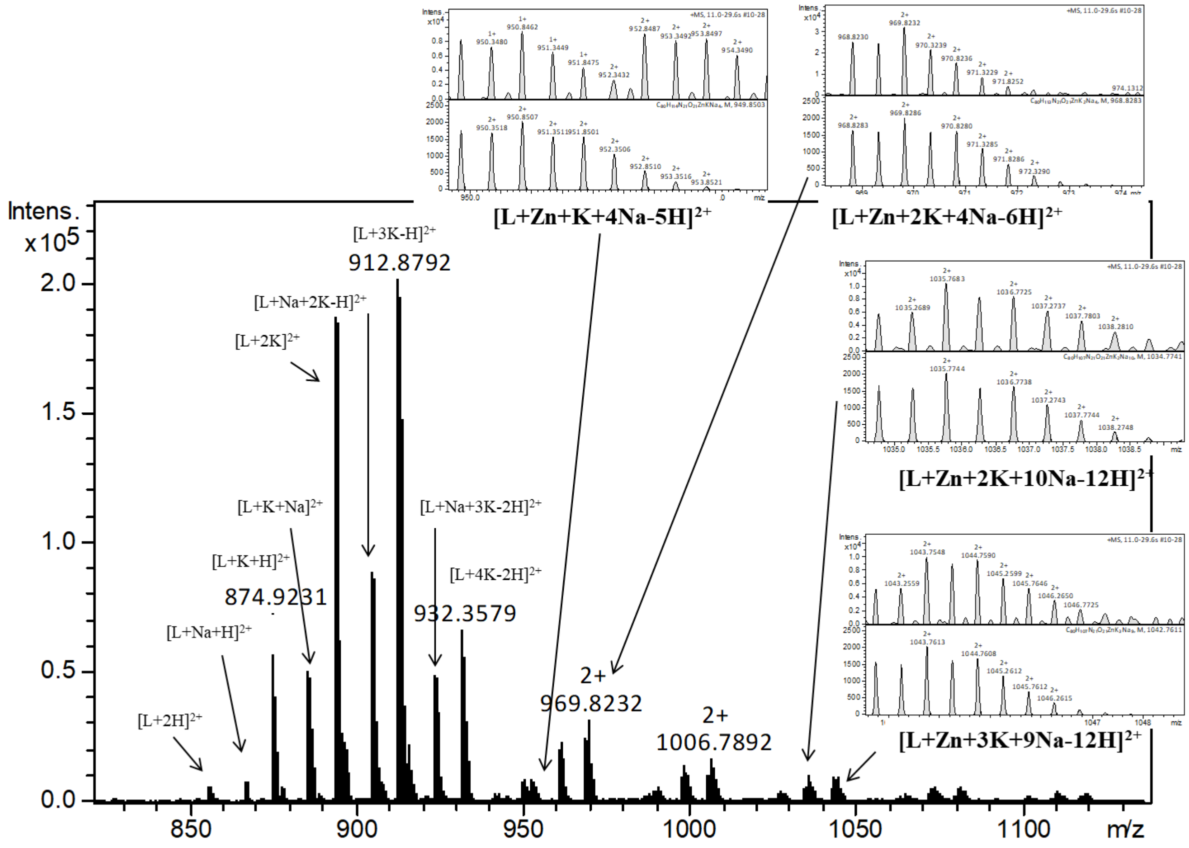

Figure S5. ESI-MS data for Zn(II) complexes of L1. Enlarged pictures: experimental (higher panel) *vs* simulated (lower panel) isotopic pattern of the complexes; shown only for main species. L1 : Zn(II) (1:1) pH 9.0.

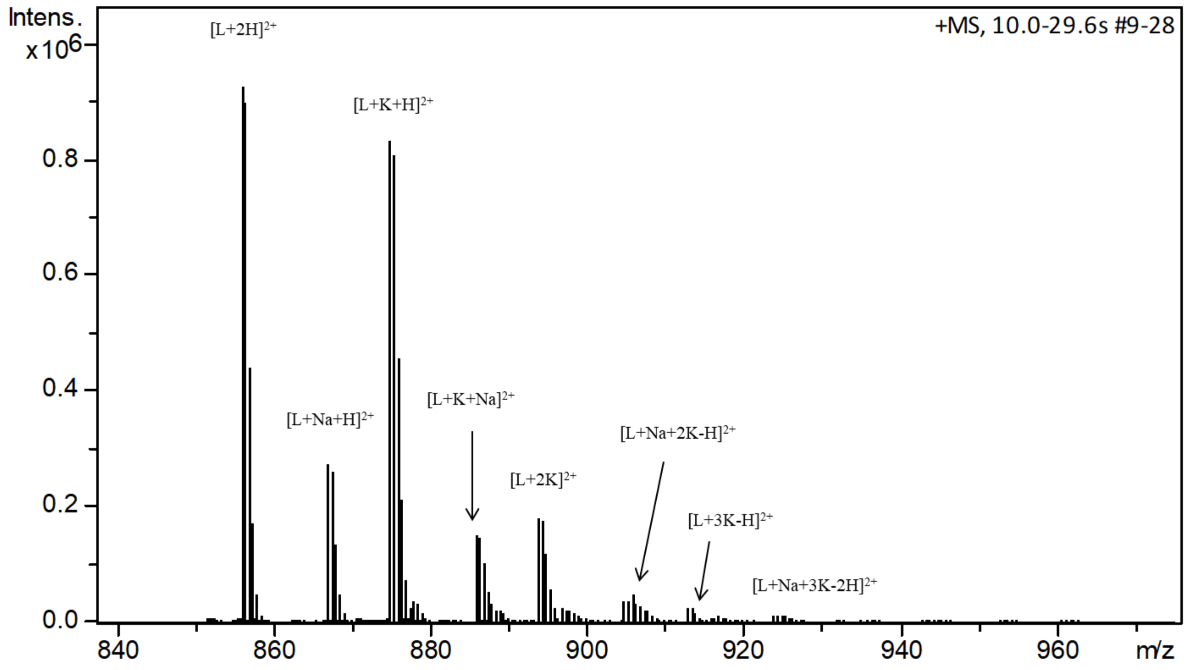

Figure S6. ESI-MS data for Zn(II) complexes of L2. Enlarged pictures: experimental (higher panel) *vs* simulated (lower panel) isotopic pattern of the complexes; shown only for main species. L2 : Zn(II) (1:1) pH 7.2.

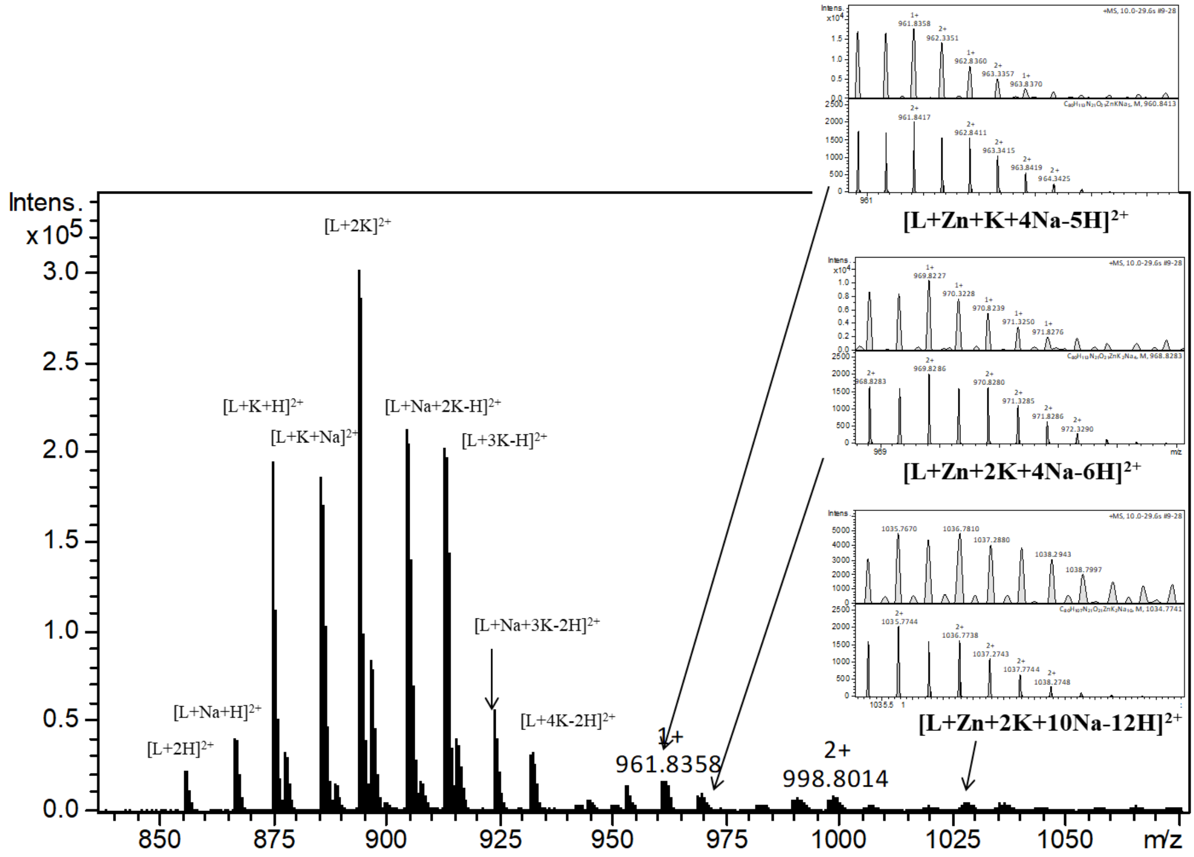

Figure S7. ESI-MS data for Zn(II) complexes of L2. Enlarged pictures: experimental (higher panel) *vs* simulated (lower panel) isotopic pattern of the complexes; shown only for main species. L2 : Zn(II) (1:1) pH 8.9.

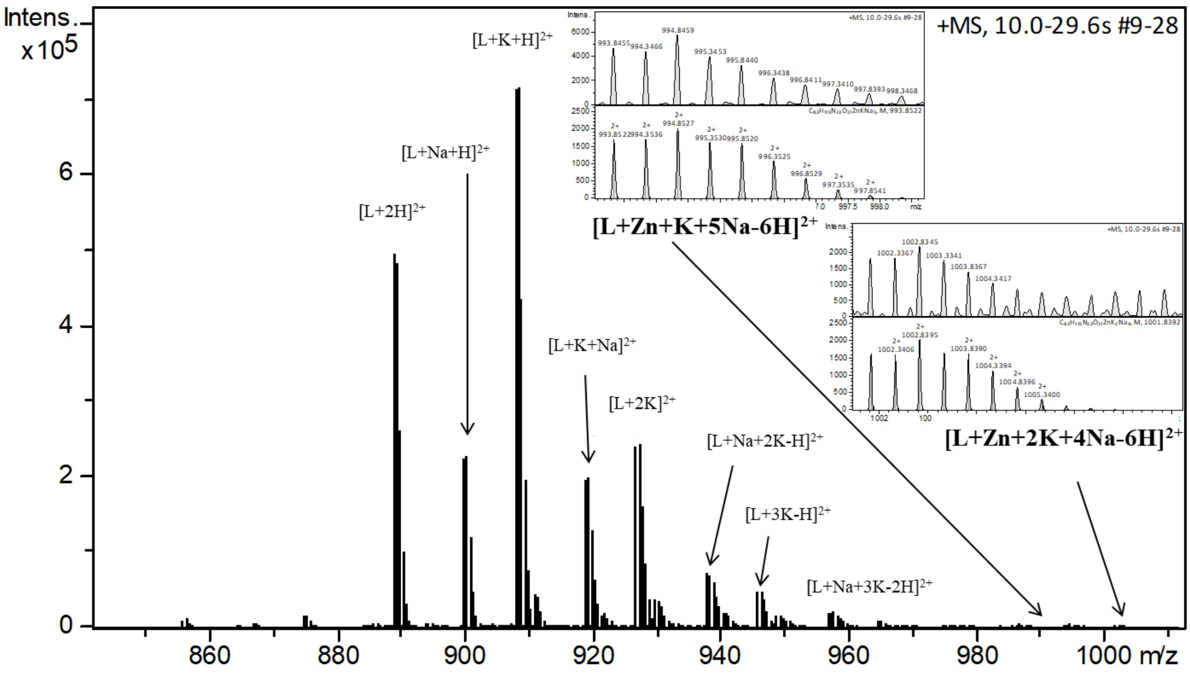

Figure S8. ESI-MS data for Zn(II) complexes of L3. Enlarged pictures: experimental (higher panel) *vs* simulated (lower panel) isotopic pattern of the complexes; shown only for main species. L3 : Zn(II) (1:1) pH 7.2.

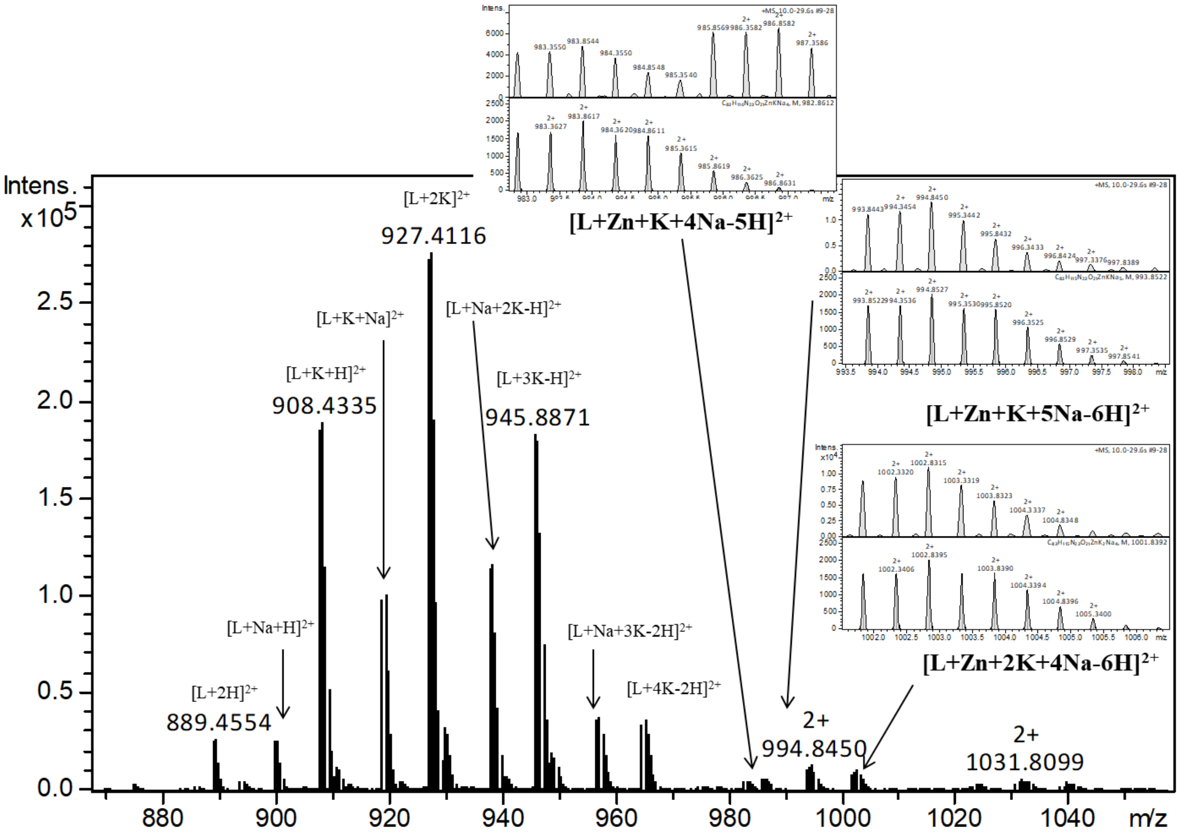

Figure S9. ESI-MS data for Zn(II) complexes of L3. Enlarged pictures: experimental (higher panel) *vs* simulated (lower panel) isotopic pattern of the complexes; shown only for main species. L3 : Zn(II) (1:1) pH 9.0.

Table S5. Experimentally measured molar extinction coefficients (ε) and absorbance maxima (λmax) for L1, L2 and L3 which concentration was determined by UV-Vis. Solvent HEPES buffer (50 mM. I = 0.16 M).Path length = 1 cm.T = 25°C.

	λ_{max} [nm]	ε [$M^{-1}cm^{-1}$]
L1	275.5	1076
L2	280	1062
L3	275.5	1077

Table S6. Antimicrobial activity of the KSAFELPHYGLLAHQ (L1), KSHFELPAYGLLAHQ (L2) and KSHFELPHYGLLAHQ (L3) and their metal(II) complexes against pathogenic microorganisms. Values are the average of three independent experiments performed in three wells.

**Ligands/ complexes MIC (μg/ml)**	***E. faecalis*** **ATCC 29212**	***S. epidermidis*** **KCTC 1 917**	***E. coli*** **ATCC 25922**	***P. aeruginosa*** **ATCC 15422**	***C. albicans*** **ATCC 10231**
L1	248 ± 5.3	226 ± 2.7	705 ± 4.1	488 ± 4.4	746 ± 4.1
Cu(II)/L1	226 ± 2.4	180 ± 4.4	664 ± 3.2	434 ± 2.2	705 ± 6.0
Zn(II)/L1	185 ± 2.2	145 ± 2.6	609 ± 4.4	394 ± 5.3	645 ± 4.76
L2	279 ± 3.8	251 ± 2.7	712 ± 0.8	517 ± 4.8	802 ± 2.4
Cu(II)/L2	229 ± 2.4	282 ± 2.4	686 ± 2.4	493 ± 5.5	778 ± 3.2
Zn(II)/L2	214 ± 2.4	195 ± 3.8	666 ± 4.1	480 ± 1.5	708 ± 4.4
L3	428 ± 2.8	373 ± 4.6	>888	556 ± 4.4	>888
Cu(II)/L3	398 ± 5.5	350 ± 3.7	>888	522 ± 2.3	>888
Zn(II)/L3	352 ± 5.0	341 ± 0.5	>888	492 ± 3.2	>888

Table S7. *In vitro* antibacterial activity of antimicrobial compounds determined as a MIC (μg/mL); NA, not applicable; NT, microorganism is not susceptible to the antimicrobial.

**Strain** MIC (μg/mL)	*E. faecalis*	*S. epidermidis*	*E. coli*	*P. aeruginosa*	*C. albicans*	**Reference**
Ciprofloxacin	1.25	NA	0.08	0.16	NT	[33]
Gentamicin	≥2.50	NA	≥2.50	0.31	NT	[33]
Polymyxin	>16	NA	1	1	NA	[34]
Gramicidin S	6.25	NA	50	50	NA	[35]
Clavanin B	256	NA	256	NA	64	[36]
Cu(II)/Clavanin B	8	NA	256	NA	64	
Zn(II)/Clavanin B	8	NA	256	NA	128	
Clavanin D	128	NA	64	NA	16	[36]

Cu(II)/Clavanin D	128	NA	256	NA	32
Zn(II)/Clavanin D	128	NA	256	NA	64
